# Complete Oral Refusal in a Pediatric Patient Following General Anesthetic for Dental Surgery: A Case Report

**DOI:** 10.1155/crpe/2710160

**Published:** 2025-10-08

**Authors:** F. Scheepers, M-Reza Nouri, L. Scheepers

**Affiliations:** ^1^Faculty of Medicine, University of British Columbia, Vancouver V6T 1Z3, British Columbia, Canada; ^2^Faculty of Dentistry, University of British Columbia, Vancouver V6T 1Z3, British Columbia, Canada; ^3^Department of Anesthesia, University of British Columbia, Vancouver V6T 1Z3, British Columbia, Canada

## Abstract

This article presents a rare case of a five-year-old pediatric patient who refused all oral intake, including swallowing saliva, for 1 week following general anesthesia for dental surgery. The patient was hospitalized for further investigation and nutritional support. After an 8-day stay, the patient was discharged at their baseline condition, having gradually started eating with continuous encouragement. No underlying medical causes were identified. The patient had a history of anxiety before the surgery, and this case highlights the critical role of addressing anxiety in postoperative care, as well as the possibility of this behavior being a rare manifestation of avoidant/restrictive food intake disorder (ARFID).

## 1. Introduction

This case report describes a rare instance of a five-year-old pediatric patient who exhibited extreme refusal to eat or drink for approximately 1 week following anesthesia for full-mouth dental restorations. The patient experienced significant difficulty swallowing, which was most likely exacerbated by postoperative pain and anxiety. This response is notably uncommon, with no documented cases in the existing literature of such severe oral refusal following dental surgery. The case underscores the importance of considering anxiety and pain management strategies in pediatric patients undergoing restorative dentistry.

## 2. Case Report

A five-year-old pediatric patient with no medical or developmental concerns underwent general anesthesia for a dental procedure that included tooth extraction, multiple fillings, and placement of crowns. The procedure was uncomplicated, and standard anesthetic agents (propofol, fentanyl, and rocuronium) were used for IV induction, followed by nasal intubation and sevoflurane maintenance. Pre-emptive antiemetics (ondansetron and dexamethasone) and analgesics (IV ketorolac) were administered, and the anesthesia course proceeded without incident. The patient was discharged in stable condition without reports of pain, coughing, or bleeding. Mild postoperative tooth sensitivity was anticipated, which was expected to be manageable with oral analgesics.

However, 2 days following the procedure, the patient was brought to the emergency department (ED) due to a refusal to eat, drink, or even swallow saliva. The parents reported that the patient was able to swallow immediately postsurgery, but after ingesting liquid Tylenol and Advil, the patient cried out in pain and subsequently refused all oral intake. At the time of presentation to the ED, the patient was afebrile with dark urine and was observed to spit out saliva.

The clinical evaluation revealed signs of moderate dehydration (dry lips, mouth, slightly sunken eyes, and fatigue). Despite being encouraged to engage in oral rehydration therapy (ORT) by the clinical team, the patient continued to refuse oral intake. No fever, rashes, or gastrointestinal symptoms were reported. A neck soft tissue X-ray was performed to rule out any deep space infections such as a retropharyngeal abscess or epiglottitis, as well as ensuring no dislodgement of dental materials. Routine bloodwork showed no significant abnormalities, including a normal white blood cell count and negative blood cultures.

The patient was admitted to the clinical teaching unit (CTU) for management of dehydration secondary to pain from the dental procedure. Upon admission, the most likely diagnosis was postoperative pain and inflammation leading to avoidance of swallowing. IV fluids were administered at full maintenance, and acetaminophen was prescribed for pain management, while oral intake was actively encouraged.

Consultations were sought from anesthesiology, ENT, and dentistry. ENT findings were unremarkable, and the team suggested that the patient's reluctance to swallow was likely driven by pain and anxiety. Dentistry concurred with this assessment, attributing the patient's symptoms to postoperative discomfort and anxiety. Anesthesia considered NSAID-induced pharyngitis as a potential diagnosis, while a comprehensive approach involving psychology, speech-language pathology, occupational therapy, and dietetics was initiated to support the patient's oral intake and emotional well-being. Psychological support focused on psychoeducation around fear and anxiety in addition to exposure therapy to gradually introduce triggers.

During assessments by psychologists and child life specialists, it was discovered that this patient had a history of situational anxiety, as reported by her parents and significant fear regarding the operation.

Despite extensive efforts, including parental and child life specialist encouragement, the patient's refusal to eat or drink persisted. A nasogastric (NG) tube was placed for enteral nutrition. ENT performed a nasopharyngoscopy, which revealed a normal oropharynx and hypopharynx, without edema or other concerning findings. The patient's gag reflex was intact, and no nerve injury was noted.

The patient's condition gradually improved over several days with continued nutritional support, encouragement to sip liquids, and psychological support. By the eighth hospital day, the patient was eating, drinking, and speaking normally. The NG tube was removed, and the patient was discharged in stable condition. A referral was made to a local pediatrician for continued monitoring. The patient has been followed at their pediatric dentist office since discharge with no complications or issues with dental treatment following their comprehensive dental restorations.

## 3. Discussion

This case represents an extremely rare clinical scenario of complete refusal to swallow, including refusal to even swallow saliva, following comprehensive dental rehabilitation in a pediatric patient. The literature contains limited references to children refusing oral intake postoperatively, but no reports have described such a severe manifestation following a dental procedure.

Previous studies have identified a strong link between preoperative anxiety and heightened postoperative pain in pediatric patients [1]. Children with high levels of preoperative anxiety often require higher doses of analgesics and have a higher risk of adverse outcomes, including prolonged hospital stays and maladaptive postsurgical behaviors [1]. A 2013 systematic review found a significant correlation between preoperative anxiety and increased postsurgical pain, which may also contribute to longer recovery times and greater consumption of pain medications [2].

Additionally, some models of chronic postoperative pain, such as the fear avoidance model, may help explain this patient's presentation [1]. According to this model, fear of pain can perpetuate avoidance behaviors, leading to a cycle of increasing pain sensitivity, avoidance of necessary actions such as swallowing, and functional disability [1]. Although this model typically applies to chronic pain, its principles are also relevant in cases of acute pain and anxiety, such as this patient. This patient may have developed a fear-avoidance cycle following the initial pain experience with swallowing, which perpetuated the refusal to eat or drink.

It is also worth considering whether this case might represent a unique presentation of avoidant/restrictive food intake disorder (ARFID). This condition is characterized by an avoidance or restriction of food intake due to fears of adverse consequences, including pain [3]. ARFID has been associated with anxiety disorders, with studies suggesting that up to 60% of patients with ARFID have a comorbid anxiety disorder [4]. Although ARFID is not typically triggered by acute postsurgical pain, this case could represent an atypical presentation where the fear of pain from swallowing led to severe food avoidance behaviors.

The role of preoperative anxiety in postsurgical outcomes is well documented [1, 2, 5, 6]. Screening for anxiety prior to surgery, particularly in pediatric dental patients, may help identify those at higher risk for complications such as excessive pain and functional impairment. Early involvement of psychological support services may also improve outcomes by addressing the underlying anxiety and fear.

## 4. Conclusion

This case highlights the potential for severe functional disability following comprehensive dental restorations in children, manifesting as an extreme refusal to swallow even saliva. The interplay between postoperative pain and anxiety can result in significant complications, including dehydration and prolonged hospitalization. Given the rarity of such cases, this report emphasizes the importance of considering baseline anxiety levels and providing comprehensive psychological support to manage postoperative anxiety and pain in pediatric surgical patients. Furthermore, it is imperative for clinicians to remain in contact with the parents in the first few days postoperatively to ensure that complications are managed effectively and in a timely manner. In this case, supportive care to keep the patient hydrated and fed adequately played a significant role until the patient resumed a normal diet. Future studies and guidelines may benefit from further exploration of preoperative anxiety screening and interdisciplinary care, as well as close postoperative follow-up, in the management of pediatric postsurgical recovery.

## Data Availability

Data sharing is not applicable to this article as no datasets were generated or analyzed during the current study.
